# Distinct composition and metabolic functions of human gut microbiota are associated with cachexia in lung cancer patients

**DOI:** 10.1038/s41396-021-00998-8

**Published:** 2021-05-17

**Authors:** Yueqiong Ni, Zoltan Lohinai, Yoshitaro Heshiki, Balazs Dome, Judit Moldvay, Edit Dulka, Gabriella Galffy, Judit Berta, Glen J. Weiss, Morten O. A. Sommer, Gianni Panagiotou

**Affiliations:** 1 Leibniz Institute for Natural Product Research and Infection Biology—Hans Knöll Institute, Jena, Germany; 2 National Koranyi Institute of Pulmonology, Budapest, Hungary; 3 School of Biological Sciences, The University of Hong Kong, Kadoorie Biological Sciences Building, Hong Kong, China; 4 County Hospital of Torokbalint, Torokbalint, Hungary; 5 MiRanostics Consulting, Oro Valley, AZ USA; 6 Novo Nordisk Foundation Center for Biosustainability, Technical University of Denmark, Kongens, Denmark; 7 Department of Microbiology, Li Ka Shing Faculty of Medicine, University of Hong Kong, Hong Kong, China

**Keywords:** Microbiome, Metagenomics, Next-generation sequencing, Metabolomics

## Abstract

Cachexia is associated with decreased survival in cancer patients and has a prevalence of up to 80%. The etiology of cachexia is poorly understood, and limited treatment options exist. Here, we investigated the role of the human gut microbiome in cachexia by integrating shotgun metagenomics and plasma metabolomics of 31 lung cancer patients. The cachexia group showed significant differences in the gut microbial composition, functional pathways of the metagenome, and the related plasma metabolites compared to non-cachectic patients. Branched-chain amino acids (BCAAs), methylhistamine, and vitamins were significantly depleted in the plasma of cachexia patients, which was also reflected in the depletion of relevant gut microbiota functional pathways. The enrichment of BCAAs and 3-oxocholic acid in non-cachectic patients were positively correlated with gut microbial species *Prevotella copri* and *Lactobacillus gasseri*, respectively. Furthermore, the gut microbiota capacity for lipopolysaccharides biosynthesis was significantly enriched in cachectic patients. The involvement of the gut microbiome in cachexia was further observed in a high-performance machine learning model using solely gut microbial features. Our study demonstrates the links between cachectic host metabolism and specific gut microbial species and functions in a clinical setting, suggesting that the gut microbiota could have an influence on cachexia with possible therapeutic applications.

## Background

Cachexia is a multifactorial disorder frequently observed in cancer patients, characterized by weight loss, muscle wasting, adipose tissue changes, physical dysfunction, and appetite loss (anorexia) induced by inflammation and abnormal metabolism [1]. The presence of this syndrome limits the treatment options for cancer patients and leads to a decrease in the quality of life (QOL) and survival [2]. Cachexia presents to varying degrees in cancer patients depending on the cancer type [3], with the highest incidences in gastrointestinal (80%) [4] and lung (60%) cancer patients [5]. Although the underlying etiology of cachexia is not fully understood, cytokine physiology has been suggested in both mouse models and humans. A decrease in anabolic factor insulin-like growth factor-1 and the increase in inflammation-related catabolic factors such as interleukin (IL-6), interferon-gamma (IFN-γ), and tumor necrosis factor-alpha (TNF-α) was observed in cachectic patients [6]. In addition, the level of lipopolysaccharide-binding protein (LBP) in serum has been suggested as a biomarker of cachexia [7].

Several approaches have been proposed in the treatment of cancer cachexia, including targeting catabolic factors, appetite stimulation, and muscle regeneration; however, these have had limited salutary effects [8]. Nutrients such as omega-3 fatty acids (eicosapentaenoic acid and docosahexaenoic acid) to mitigate inflammation, and leucine and milk proteins to promote protein synthesis have also been suggested as potential treatment options [9]. However, no single therapeutic approach is sufficient to treat this multifactorial disorder, and multimodal therapy considering nutrition, exercise, and pharmacological agents are likely needed [8, 10].

The gut microbiota is gaining attention as a new target for cachexia treatment, due to its critical role in providing depleted nutrients, modulating gut hormones, cachexia-related cytokines, and improving gut barrier function [11]. Furthermore, the gut microbiota has been associated with different disorders including those that share symptoms with cachexia, such as anorexia [12], malnutrition [13], and chronic fatigue syndrome (CFS) [14]. A recent study from Potgens et al. has investigated gut microbiota in cachectic mice with colon carcinoma and linked cachexia successfully with *Klebsiella oxytoca*, a specific gut bacterial species involved in altering gut barrier function [15]. From the aspect of reversing cancer cachexia, a particular strain, *Faecalibacterium prausnitzii A2–165 (DSM 17677)*, has been used in cachectic mice with colon carcinoma. However, it did not modify the gut permeability, and no biomarkers of gut barrier function were altered [7]. Notably, most studies to date are limited to murine models with colon cancer, neuroblastoma, or leukemia, and the analytical approaches to disentangle microbiome composition were all based on 16S rRNA gene sequencing, a less informative or sensitive methodology in comparison to shotgun metagenomic sequencing [7, 15–18].

Here, we performed an in-depth analysis of the plasma metabolome, the gut bacterial taxonomy, and functionality in 31 human lung cancer patients by applying untargeted metabolomics to patient plasma samples and shotgun metagenomics to collect stool samples. Specific metabolites, intestinal microbial species, and their metabolic pathways were associated with cachexia status. In order to get a comprehensive picture of the role of the gut microbiome in cachexia, we subsequently integrated the taxonomic and functional signatures with metabolomics data. A machine learning classifier of cachectic and non-cachectic patients, with the combinatorial effect of microbiota features taken into account, was also developed and further supported the putative role of gut microbiota. Here we aim to identify the microbiome associations with cachexia to open up the way for new therapeutic options for this critical medical condition that influences cancer treatment outcomes.

## Results

### Cachexia affect the survival probability of lung cancer patients

**Fig. 1:**
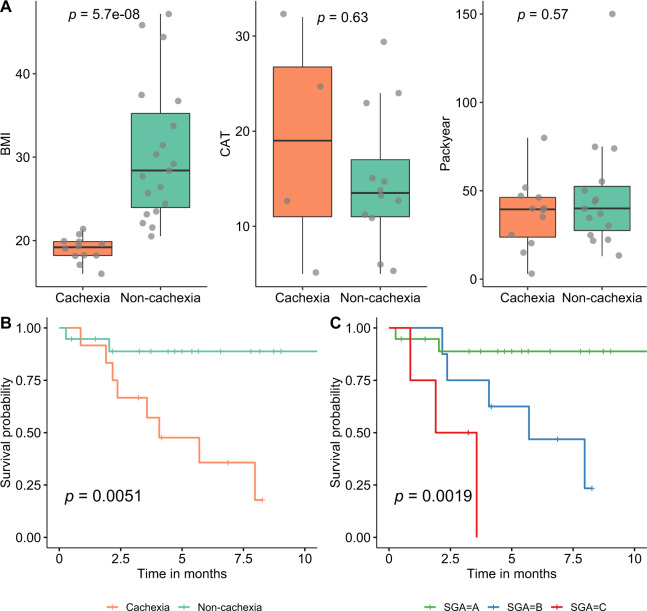
Comparison of clinicopathological characteristics and cancer cachexia. **A** Comparisons of body mass index (BMI), COPD assessment test (CAT), and Pack-year (calculated by multiplying the number of packs of cigarettes smoked per day by the number of years the person has smoked) between cachexia and non-cachexia. **B**, **C** Survival analysis highlights the impact of cachexia on the overall survival of cancer patients, according to cachexia (**B**) and SGA grouping (**C**) (*p* = 0.0051 and *p* = 0.0019, respectively, Log-rank test).

Thirty-one lung cancer patients, 12 women, and 19 men were enrolled at the National Koranyi Institute of Pulmonology (Budapest, Hungary) and at the County Hospital of Torokbalint (Torokbalint, Hungary). The patients were classified as A (well-nourished, scores 0–4, *n* = 19), B (moderately or suspected of being malnourished, scores 5–9, *n* = 8), or C (severely malnourished, scores >9, *n* = 4) based on the abridged Patient-Generated Subjective Global Assessment (aPG-SGA) [19]. We merged groups B and C (aPG-SGA scores >4), referred to herein as the cachexia group (*n* = 12), whereas patients classified as A served as the non-cachexia group (aPG-SGA scores 0–4, *n* = 19). There were no significant differences in the distribution of four lung cancer subtypes or cancer stage between the two groups (*p* > 0.05 for both subtype and stage, Fisher’s exact test, Supplementary Table S1). Moreover, the two groups are matched in age, sex, and other parameters that could potentially affect the gut microbiota composition (Supplementary Table S2). As expected, the cachexia group has a significantly lower body mass index (BMI) compared to non-cachectic patients (*p* = 5.7e−08, Wilcoxon rank-sum test) (Fig. 1A). Univariate survival analysis demonstrated that the cachexia patients have significantly lower survival probability (vs. non-cachexia, *p* = 0.0051, Log-rank test) (Fig. 1B); furthermore, there were significantly increased survival in patients with SGA scores A compared to B or C, (*p* = 0.0019, Log-rank test) (Fig. 1C).

### The lower level of plasma BCAAs in cachexia

**Fig. 2:**
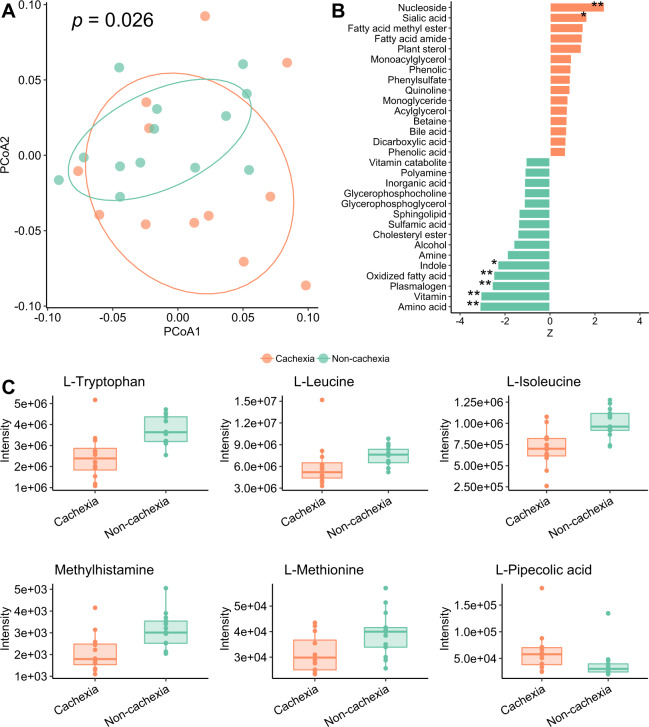
Altered plasma metabolome profiles in cachexia. **A** Principal coordinate analysis (PCoA) plot of cachexia and non-cachexia patient groups based on plasma metabolomic profiles (Bray–Curtis distance) (*p* = 0.026, *r* = 0.110, ANOSIM). **B** Differentially abundant metabolite classes (*p < 0.05, **p < 0.01, Wilcoxon rank-sum test). Orange: higher abundance in cachexia; green: lower abundance in cachexia group. **C** Plasma amino acids with significant differences between cachexia and non-cachexia patient groups (*p* < 0.05, Student’s *t*-test).

To characterize the plasma metabolites profile in cachexia in a clinical setting, we collected plasma samples from our patients that were subject to untargeted metabolomics analysis utilizing ultra-high-performance liquid chromatography-quadruple time-of-flight mass spectrometry (UHPLC–QTOF–MS). In total, more than 5000 metabolite features were captured, of which 314 common metabolites were identified in a semi-targeted manner. Multi-variate statistical analysis shows that the metabolomic profiles of cachexia and non-cachexia patient groups (based on Bray–Curtis dissimilarities) are statistically significantly different (*p* = 0.026, *r* = 0.110, ANOSIM), with comparatively scattered cachectic samples observed, suggesting higher variability in the cachectic patients (*p* < 0.01, betadisper) (Fig. 2A). The increase of dispersion has been observed in different diseases, such as colorectal cancer, Crohn’s disease, and liver cirrhosis [20]. The metabolite classes, such as amino acids, vitamins, and indoles were significantly depleted in cachectic lung cancer patients (Fig. 2B).

In total, 41 individual metabolites were identified as differentially abundant between the two groups (*p* < 0.05, Student’s *t*-test) (Fig. S1), with two of the identified metabolites including isoleucine, remained significant after multiple hypothesis testing corrections (false discovery rate (FDR)-corrected *p* < 0.2). Overall, essential amino acids, such as isoleucine, leucine, and tryptophan were significantly more abundant in non-cachectic patients (Figs. 2C and S2). Low serum cholesterol level has been previously suggested as a biomarker for malnutrition [21]. In our data, there was no difference in serum cholesterol level between the two groups (*p* = 0.774, Student’s *t*-test). Consistent with leucine and isoleucine, another member of branched-chain amino acids (BCAAs), valine, was also found in a lower amount in cachectic patients, but was not statistically significant (*p* = 0.103, Student’s *t*-test). Of note, the depletion of plasma BCAAs has also been shown in children with severe kwashiorkor (malnutrition caused by a lack of protein in the diet). In contrast, an increase in plasma BCAAs levels has been observed in type 2 diabetes (T2D) and obese subjects compared to healthy people [22], highlighting the high relevance of plasma BCAAs to metabolic balance. Accordingly, the use of leucine in the representation of BCAAs has been previously suggested as dietary supplementation for tackling cachexia [9]. In comparison, pipecolic acid, a non-proteogenic cyclic amino acid produced during the degradation of lysine, was the only amino acid significantly enriched in our cachectic patients (*p* < 0.05, Student’s *t*-test). This may result from the excessive degradation of lysine due to increased protein degradation and decreased protein synthesis. The level of pipecolic acid has been reported to be elevated in patients with liver cirrhosis and hepatocellular carcinoma [23].

In summary, untargeted metabolomics revealed key circulating plasma metabolites in cachectic lung cancer patients that may have potential clinical relevance in cachexia syndrome development or progression. Alteration of blood metabolites might be associated with gut microbiota and their metabolic pathways, as demonstrated before [24].

### Cancer cachexia patients have a distinct gut bacterial profile

**Fig. 3:**
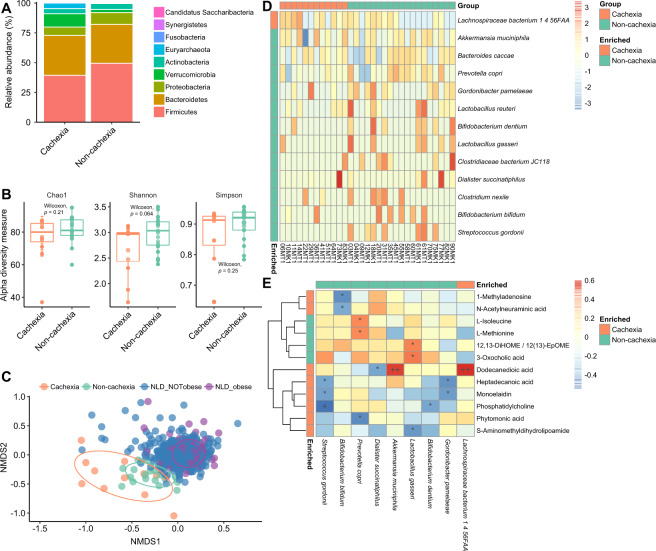
Distinct gut microbiota composition between cachexia and non-cachexia patients. **A** Phylum abundance comparison between cachexia and non-cachexia patient groups. **B** Comparison of microbial alpha diversity: Chao1 (*p* = 0.21, Wilcoxon rank-sum test), Shannon index (*p* = 0.064, Wilcoxon rank-sum test), Simpson index (*p* = 0.25, Wilcoxon rank-sum test). **C** Non-metric multidimensional scaling (NMDS) plot comparing cachexia and non-cachexia patient groups together with a healthy Dutch cohort (NLD) of 471 subjects, based on the gut bacterial species compositions using Bray-Curtis dissimilarities (*p* = 0.001, *r* = 0.212, ANOSIM). The BMI cutoff of 25 was used to group NLD samples into “NLD_NOTobese” and “NLD_obese”. **D** Heatmap of differential abundant bacterial species (*p* < 0.05, prevalence higher than 20%). Color scale represents the row-scaled log-transformed relative abundances of species. **E** Potential mechanistic links between cachexia-associated gut microbiota species and serum metabolites. Spearman’s rank correlations were calculated between differentially abundant species and differentially abundant metabolites (*p < 0.05, **p < 0.01, ***p < 0.001, +FDR < 0.1, ++FDR < 0.05, +++FDR < 0.01, Spearman’s rank correlation).

Next, we analyzed the change of gut microbiome according to cancer cachexia using 31 fecal samples collected from our lung cancer patients. Bacterial DNA was isolated from the fecal samples and used for shotgun metagenomic sequencing at an average depth of 6 Gbp. We compared the gut microbiome composition between cachexia and non-cachexia and observed no differentially abundant phyla between the two groups (Fig. 3A). Regarding microbiota community diversity, no significant difference was observed in alpha-diversity between cachexia and non-cachexia patients (*p* > 0.05, Wilcoxon rank-sum test) (Fig. 3B). However, significantly different microbiota composition based on Bray-Curtis dissimilarities was observed between the two groups (*p* = 0.001, *r* = 0.247, ANOSIM) (Fig. 3C), as well as the taxonomic evaluation of dispersion (*p* < 0.001, betadisper). No significant associations were found between overall microbiota compositions and cancer stage in our cohort (*p* > 0.05, PERMANOVA). Subsequently, we compared the bacterial species composition of our lung cancer samples with a large healthy European cohort (*n* = 471, Dutch) [25]. The cachexia group was placed distinctly from other groups (non-cachexia cancer patients or healthy individuals) in the ordination plot (*p* = 0.001, *r* = 0.212, ANOSIM) (Fig. 3C). The dissimilarity between the cachexia group and healthy lean people also reflected the complexity of the gut microbiota structure of cachexia patients rather than merely resembling that of lean people. We assessed the *Firmicutes*/*Bacteroidetes* (F/B) ratio which has been hypothesized to be lower in cachexia patients due to its reported association with obesity and BMI [26, 27], but observed no significant difference between the cachexia and non-cachexia groups (*p* = 0.1196, Wilcoxon rank-sum test), or between obese patients (BMI > 30) and non-obese patients (BMI < 30) in our cohort (*p* = 0.4113, Wilcoxon rank-sum test). Moreover, no significant positive correlation was found between the F/B ratio and BMI in our lung cancer cohort (*p* = 0.5747, *rho* = 0.1044, Spearman’s rank correlation).

Next, we focused on comparisons at the species level and identified fifty-one differentially abundant bacterial species between the two groups (*p* < 0.05) (Fig. S3), most of which (*n* = 44) remained significant after multiple hypothesis testing corrections (FDR-corrected *p* < 0.05). A total of 13 significant species (Fig. 3D) were also prevalent (higher than 20%) among all patients, the vast majority of which were more abundant in the non-cachexia group. *Prevotella copri* showed significantly lower abundance in cachectic patients (FDR-corrected *p* = 0.006), in which the depletion of plasma BCAAs was observed. Notably, *P. copri* has been associated with enhanced gut microbiota biosynthesis and circulating levels of BCAAs [28]. *Klebsiella oxytoca*, a species previously associated with cancer cachexia in mice [15], was found to be significantly higher in lung cancer patients with cachexia (*p* = 0.013, FDR = 0.052), though with low prevalence in this human-based cohort. Next, we analyzed *Faecalibacterium prausnitzii*, a gut bacterium with anti-inflammatory and gut barrier-enhancing properties [29, 30], which as a treatment option did not improve the gut permeability or the gut barrier function of cachectic mice [7]. Importantly, in our human study, *F. prausnitzii* was significantly more abundant in non-cachectic patients, though detected only by Wilcoxon rank-sum test (*p* < 0.05). Further strain-level analysis for *F. prausnitzii* showed that another strain *M21/2* had a higher difference between non-cachexia and cachexia patients (*p* = 0.101, Wilcoxon rank-sum test), as compared with the strain *A2-165* investigated before [7], suggesting the potential of alternative strains in future treatment (Fig. S4).

Previous studies have revealed the considerable effects of gut microbiota on blood metabolite profiles [24, 31]. In our lung cancer cohort, we also observed a significant correlation between the overall plasma metabolome and the gut microbial species (*p* = 2e−04, *r* = 0.4565, Mantel test). To further disentangle the interplay between gut microbiota taxonomy and plasma metabolite pool, we correlated the significantly differential abundant microbial species and metabolites between cachexia and non-cachexia patients. The plasma level of isoleucine, a member of BCAAs, was significantly positively correlated with the abundance of *P. copri* (Fig. 3E), as demonstrated before [28]. The 3-oxocholic acid was more abundant in non-cachexia patients and had a positive correlation with gut species *Lactobacillus gasseri*, a potential probiotic [32]. Accordingly, *L. gasseri* was also enriched in the non-cachexia group (vs cachexia, *p* = 0.021, FDR = 0.082) (Fig. 3D). These results further support the association between the gut microbiome alterations and circulating plasma metabolites that may have clinical implications in cachexia syndrome development or progression.

### Alteration of gut microbiota metabolic pathways associated with cachexia

The use of shotgun metagenomic sequencing also enabled us to further examine the variation of gut microbiota functions according to cachexia in lung cancer patients. Using the MetaCyc pathway abundances based on UniRef90 gene annotation results, we observed no significant differences of functional alpha diversity between cachectic and non-cachectic patients (*p* = 0.48 and *p* = 0.86, Shannon and Simpson index, respectively, Wilcoxon rank-sum test). In contrast, we found a significant difference in microbial community functional profiles (Bray-Curtis dissimilarities calculated from MetaCyc pathway abundances) between the two patient groups (*p* = 0.035, *r* = 0.129, ANOSIM), but not for the functional evaluation of dispersion (*p* = 0.068, betadisper).

**Fig. 4:**
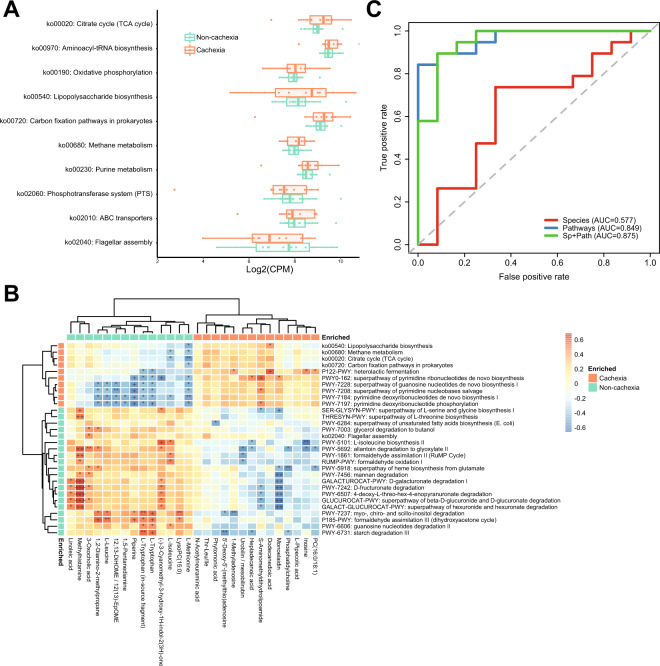
Functional change of gut microbiota according to cancer cachexia. **A** Significantly enriched or depleted microbial KEGG pathways from pathway enrichment analysis. Pathways in orange: cachexia-enriched; green: cachexia-depleted. CPM: copies per million. **B** Heatmap of Spearman’s rank correlation analysis between significantly enriched pathways versus differentially abundant metabolites (*p < 0.05, **p < 0.01, ***p < 0.001, +FDR < 0.1, ++FDR < 0.05, +++FDR < 0.01, Spearman’s rank correlation). Both differentially abundant MetaCyc pathways and significant KEGG pathways from enrichment analysis were used here. **C** Receiver operating characteristic (ROC) curve plots of Random Forest models based on the gut microbial taxonomic and pathway features of cancer patients for differentiating lung cancer patients with and without cachexia.

By directly comparing the overall abundances of pathways, we found that catabolic pathways of certain complex carbohydrates (starch, mannan) and sugar derivatives (glucuronide, fructuronate, myo-, chiro- and scillo-inositol), as well as anabolic pathways of several amino acids, were significantly lower in the cachexia patient group compared to the non-cachexia group (*p* < 0.05, Wilcoxon rank-sum test) (Fig. S5). Such decreased gut microbiota biosynthesis of amino acids under cachexia, including isoleucine, threonine, serine, and glycine, is in agreement with our plasma metabolomics-based finding aforementioned, especially for BCAAs. Next, we performed KEGG pathway enrichment analysis using GAGE [33], an approach that identifies concordant changes of the genes present in a particular pathway. As a result, purine and methane metabolism pathways were enriched in the cachexia group (Fig. 4A). In line with our findings, the alteration of purine metabolism has been observed in the gut microbiota of humans after body weight loss induced by Roux-en-Y gastric bypass, as well as in the comparison between older and younger people that have different muscle mass [34]. Methane may reduce appetite by direct stimulation of intestinal hormone glucagon-like peptide-1 (GLP-1) [35]. Of note, the methanogen *Methanobrevibacter smithii* was identified as the signature species of our cachectic group (*p* < 0.05, IndVal test) and has been associated with anorexia, metabolic abnormalities [36], and chronic constipation [37]. In addition, heterolactic fermentation, which was found enriched in cachectic patients (Fig. S5), might be highly relevant to methanogenesis, as lactate is the most favorable substrate for methanogens [38]. To further assess the credibility of our pathway analysis, we next investigated functions with known involvement in cachexia. A recent study has identified LBP in the serum to be a new biomarker of cancer cachexia [7]. Lipopolysaccharide (LPS) is a type of proinflammatory bacterial compound that can cause reduced intestinal barrier function and increase its translocation upon gut barrier alteration [39], as well as inducing muscle catabolism mediated by toll-like receptor 4 (TLR4) [40]. Our analysis confirmed the significant enrichment of the microbiota LPS biosynthesis pathway in the cancer cachectic patients versus non-cachectic patients (*p* < 0.05) (Fig. 4A).

Given the differential abundances of specific carbohydrate degradation pathways, we then compared the abundances of carbohydrate-active enzymes (CAZy) in the two patient groups. At a high level in the CAZy hierarchy, all CAZy classes were more abundant in the cachexia group (Fig. S6), although not statistically significant (*p* > 0.05, Wilcoxon rank-sum test). Across all samples, 439 CAZy families were detected. Twenty-nine CAZy families, including 18 enriched and 11 depleted in cachectic patients, were found significantly differentially abundant (*p* < 0.05, Wilcoxon rank-sum test), the majority of which belong to the glycoside hydrolases class.

We have identified several microbial species to be correlated significantly with differential abundant plasma metabolites (Fig. 3E). Interestingly, the functional pathways of gut microbiota were found to have more and stronger correlations with those metabolites than microbial species did (Fig. 4B). This implies that the influence of gut microbiota on the plasma metabolites profile was more likely through the combinatorial effects of multiple bacteria, or microbial consortium, rather than individual microbial species. Furthermore, it highlights the importance of microbiota functions in interacting with plasma metabolome and affecting host phenotypes, including cachexia. Additionally, the positive correlation between plasma level of L-isoleucine and the gut microbial pathway “PWY-5101: L-isoleucine biosynthesis II” (*p* = 0.010, *rho* = 0.509, Spearman’s rank correlation) suggests the impact of the biosynthesis of amino acids in the gut to the plasma amino acid levels. The plasma level of methylhistamine was correlated with a range of gut microbiota pathways and significantly enriched in the non-cachectic patient group (Fig. 4B). Similarly, the plasma levels of vitamins, such as B vitamins pyridoxal and pyridoxamine (both lower in a cachectic group), also showed multiple correlations with microbiota functions and species (Fig. S7).

In summary, our results highlight the distinct gut microbiota functional capacity in cachectic patients and the close relationship between gut microbial functions and the plasma metabolites in cachexia.

### Gut microbiota features as a proxy of cachexia status

Next, we built a fivefold cross-validation Random Forest classification model using microbiota-derived features to further test the associations between the human gut microbiome and cachexia in a clinical setting. We also sought to verify the relevance and robustness of identified differentially abundant taxa and pathways. In our European cohort (non-cachexia [*n* = 19], cachexia [*n* = 12]), the AUC of the model based on differentially abundant species (*n* = 51) was only 0.577, while using the differentially abundant MetaCyc pathways as features (*n* = 27) improved the performance (AUC = 0.849) (Fig. 4C). With combined features of species (*n* = 51) and MetaCyc pathways (*n* = 27), the AUC reached 0.875. This high-performance machine learning model, which takes into account the complex microbial interactions, further suggests the essential role of gut microbiota in cachexia development. This model has helped to identify a group of microbial species and functions (Fig. S8) whose combinatorial effects may be associated with cachexia in lung cancer patients. Presumably, a simplified model where a single bacterium can have a profound effect on cachexia status may not be sufficient, considering the high complexity behind this disorder. This complexity might be a possible reason why the supplementation of *Faecalibacterium prausnitzii* for cachexia treatment in mice was not successful [7]. The hybrid model had a moderate performance (AUC = 0.7) when applied to an independent validation cohort of seven lung cancer patients (non-cachexia [*n* = 5], cachexia [*n* = 2]) recruited in a US clinic, which could also be attributed to microbiome differences between the US and our European patients independently of cachexia status.

## Discussion

Cancer cachexia is associated with worse performance status and frequently limits oncotherapy administration. To date, we have no effective therapy to prevent cachexia. Using 16S rRNA sequencing, a less advanced technique compared to shotgun metagenomics used in our work, recent studies have shown the involvement of the microbial community in cachexia by analyzing the gut microbiome in murine models of colon cancer and leukemia [7, 18]. Here, we performed shotgun metagenomic sequencing and plasma untargeted metabolomics in a cohort of cancer patients, aiming to identify gut bacteria species and metabolic functions that are associated with cachexia. Through a comprehensive and integrative analysis of these omics data, we disentangled multiple links among gut microbial species, functions, and plasma metabolites, which may collectively and ultimately contribute to the development of this complex and multimodal disorder. Our findings not only provide clinical evidence between gut microbiota and cachexia but also confirm the previous results from preclinical animal models. The contribution of the gut microbiome in cachexia was further evaluated by a machine-learning model taking into account the complex combinatorial effects of gut microbiota features. This model achieved high accuracy in discriminating patients with different cachexia status in the training cohort and acceptable performance in a small independent validation cohort.

Despite our increased understanding of cachexia, previous work into gut microbiota was based on mice models that cannot fully recapitulate human cancer cachexia [41]. The lack of appropriate mice models and the differences in the complexity of the human and mice metabolic pathways hindered the more in-depth investigation and the effect of potential intervention. Very recently, Talbert et al. [41] have developed a mouse model named KPP that can better model the cachexia experienced by cancer patients. This model can be used to further validate our findings in the future, or even with another model that more closely resembles the cachectic lung cancer patients. A possible limitation of our study is that we included only lung cancer patients. The relatively low sample size might also contribute to the non-significant difference in *Firmicutes/Bacteroidetes* ratio. A very recent study has demonstrated poor similarity in gut microbial taxonomic abundances between humans and mice after fecal transplantation [42]. This further highlights the importance of investigating the relationship between cancer cachexia and gut microbiota in clinical cohorts of larger sample sizes. Despite that, our investigation into gut microbiota and plasma metabolome in cachectic lung cancer patients were able to achieve consistent findings with those preclinical studies and the studies linking gut microbiota with features of cachexia such as body weight loss, low muscle mass, and low appetite [34]. Based on our cross-sectional study, a longitudinal cohort study of more power could be implemented in the future, which collects samples from the same patients before and after cachexia development, likely allowing for predicting modeling and stronger conclusions to be drawn. A limitation of using PG-SGA instead of the international consensus of cachexia [43] is that we characterize and compare patients by their nutritional status. While aPG-SGA stage A (scores 0–4) includes well-nourished patients without cachexia, stage B (scores 5–9) and C (scores > 9) may include not only patients with cancer cachexia but also moderate and severely malnourished patients. Recently, another group reported a secondary analysis of a multicenter, cross-sectional, observational study of 4231 patients with cancer [44]. They found that PG-SGA was highly specific and could be used as a tool to screen patients for cancer cachexia. In our study, we set aPG-SGA score >4 cut-offs for cachexia, which is expected to have a 90.3% sensitivity of detecting cachexia, with scores below this cutoff having a negative predictive value for excluding cachexia at 98.48% [44].

Our study offers a snapshot of gut microbiota and plasma metabolome alteration in lung cancer patients with cachexia. To our knowledge, this is the first endeavor to investigate the role of gut microbiota in cachexia in the clinical setting. Hopefully, it will contribute to relevant clinical research and possible clinical targets in the future to attenuate, prevent or treat cachexia. Future nutritional supplements may include both amino acids and bile acids such as methylhistamine and 3-oxocholic acid. From the microbiota point of view, it might be beneficial to use treatments that can reduce gut inflammation and restore gut barrier function disrupted by increased LPS production. Previous work has tested the effect of a single bacterium identified from animal studies [45–47]. As an extension, microbial cocktails or probiotics containing mixtures of beneficial species newly identified in this study, such as *Lactobacillus gasseri* and *Prevotella copri* might be further tested in the future. Another clinical aspect is the fecal microbiota transplantation to restore healthy microbiota that might also be a possible future approach to assess the clinical importance of gut microbiota in cachexia. Lastly, combinations of different modes of therapy may be more effective due to the metabolic complexity of this disorder. Future prospective studies are needed to confirm these findings presented here.

## Methods

### Ethics statement

Our study was performed in accordance with the guidelines of the Helsinki Declaration of the World Medical Association. The national-level ethics committee (Hungarian Scientific and Research Ethics Committee of the Medical Research Council (ETTTUKEB-50302-2/2017/EKU)) officially approved the study. All patients recruited were consented to the study. The clinicopathological information was collected, then patient identifiers were removed, and afterward, patients cannot be identified either directly or indirectly.

### Study population

In total 31 lung cancer patients (12 female and 19 male) were enrolled between 2017 and 2018 at the National Koranyi Institute of Pulmonology, Budapest, Hungary, and at the County Hospital of Pulmonology, Torokbalint, Hungary (Supplementary Table S1). We included patients with histologically confirmed adenocarcinoma (ADC) (*n* = 16), squamous cell carcinoma (SCC) (*n* = 10), non-small cell lung carcinoma not otherwise specified (NSCLC-NOS) (*n* = 1), and small cell lung carcinoma (SCLC) (*n* = 4). The 58% (*n* = 18) of the patients included were diagnosed with advanced-stage disease (Stage IIIB/IV). Clinical TNM (Tumor, Node, Metastasis) stage according to the Union for International Cancer Control (8th edition) and age at the time of diagnosis were recorded. We included consecutive (in terms of BMI) patients in our study, thus representing an overall Gaussian distribution of BMI for lung cancer patients (*p* = 0.3385, Kolmogorov–Smirnov test). Patients were scored *A* (*n* = 19), *B* (*n* = 8), and *C* (*n* = 4) based on aPG-SGA [19]. The SGA scores were measured based on BMI, weight changes, food intake, symptoms of eating (appetite), and functional capacity. At the time of study conduct, PG-SGA allowed for more objective classification into three categories. Since that time, others have supported that PG-SGA is a more comprehensive and more sensitive nutritional assessment method (compared to cancer cachexia defined according to international consensus) for detecting changes in QOL domains and can contribute to the identification of QoL deterioration risk [48]. Clinicopathological data included gender, age, stage, and overall survival (OS). OS was calculated from the time of diagnosis until death or last available follow-up. The date of the last follow-up included in this analysis was February 2019. All patients had no known inflammatory bowel disease and no antibiotics usage 60 days prior to stool sample collection.

### Treatments

All treatments across all centers were conducted in accordance with contemporary National Comprehensive Cancer Network guidelines.

### Schedule of sample collection procedures

Stool and blood baseline samples were obtained at the same time point before the initiation of systemic therapy after signed informed consent was obtained. All samples were placed on the day of collection in the −80 °C freezer.

### US validation cohort information

Stool samples were collected from a human lung cancer cohort of seven individuals (Supplementary Table S3) at Western Regional Medical Center, Goodyear, Arizona, USA, after signed informed consent under a protocol approved by the Western Institutional Review Board (WIRB protocol number 20140271, Pallyup, Washington, USA). Bacterial DNA extraction, library preparation, and shotgun metagenomic sequencing followed the same approach as the EU Hungary cohort.

### Plasma metabolomic analysis

Untargeted metabolomics profiling of patient plasma samples was performed by Afekta (Kuopio, Finland), as detailed below.

#### Sample preparation

The plasma samples were prepared as follows: an aliquot of the sample, 100 μL, was mixed with 400 μL of acetonitrile and mixed by pipetting. The samples were placed on a 96-well filter plate, which was centrifuged at 700 × *g* for 5 min at 4 °C. Small aliquots were taken from each sample, mixed together in a single tube, prepared in an identical way to the other samples, and used as the quality control (QC) sample in the analysis. The fecal samples were prepared as follows: 300 μl of cold 80% aqueous methanol was added per 100 mg of sample into homogenizer tubes. The sample preparation procedures were performed on dry ice with cooled instruments. The samples were homogenized with Bead Ruptor 24 Elite (OMNI International) with Heart program (6 m/s, 30 s). Next, the samples were vortexed for 10 s and centrifuged at 13,000 rpm and 4 °C for 10 min. The supernatant was collected on a 96-well filter plate, which was centrifuged at 700 × *g* for 5 min at 4 °C. The QC sample was prepared in the same way as for the plasma samples.

#### LC-MS analysis

The samples were analyzed by liquid chromatography-mass spectrometry consisting of a 1290 Infinity Binary UPLC coupled with a 6540 UHD Accurate-Mass Q-TOF (Agilent Technologies), as described previously [49]. In brief, a Zorbax Eclipse XDB-C18 column (2.1 × 100 mm, 1.8 μm; Agilent Technologies) was used for the reversed-phase (RP) separation and an Acquity UPLC BEH amide column (Waters) for the HILIC separation. After each chromatographic run, the ionization was carried out using jet stream electrospray ionization (ESI) in the positive and negative mode, yielding four data files per sample. The collision energies for the MS/MS analysis were selected as 10, 20, and 40 V, for compatibility with spectral databases.

#### Data analysis

The data analysis was performed separately on each of the four modes and sample type combinations, resulting in a total of eight preprocessing runs. The analysis was conducted in R version 3.5.0 using in-house scripts. The untargeted metabolomics method utilized here yielded semi-quantitative data, generating the abundance of each metabolite as peak areas. Signals with too many missing values were removed by requiring a measured value in at least 60% of the samples in at least one of the study groups. The signals were corrected for the drift pattern caused by the LC-MS procedures. Regularized cubic spline regression was fit separately for each signal on the QC samples. The smoothing parameter was chosen from an interval between 0.5 and 1.5 using leave-one-out cross-validation to prevent overfitting. The performance of the drift correction was assessed using non-parametric, robust estimates of the relative standard deviation of QC samples (RSD*) and D-ratio* as quality metrics. Drift correction was only applied if the value of both quality metrics decreased, leading to enhanced quality. Otherwise, the original signal was retained. After the drift correction, low-quality signals were removed. Signals were kept if their RSD* was below 20% and their D-ratio below 40%. In addition, signals with classic RSD, RSD*, and basic D-ratio all be-low 10% were kept. This additional condition prevents the removal of signals with very low values in all but a few samples. These signals tend to have a very high value of D-ratio*, since the median absolute deviation of the biological samples is not affected by the large concentration in a handful of samples, causing the D-ratio* to overestimate the significance of random errors in measurements of QC samples. Thus, other quality metrics were applied with a conservative limit of 0.1 to ensure that only good quality signals were kept this way. Missing values were imputed using random forest imputation. Signals were then normalized using inverse-rank normalization, to approximate a normal distribution. QC samples were removed prior to imputation and normalization, to prevent them from biasing the procedures.

#### Compound identification

The chromatographic and mass spectrometric characteristics (retention time, exact mass, and MS/MS spectra) of the significantly differential molecular features were compared with entries in an in-house standard library and publicly available databases, such as METLIN and HMDB, as well as with published literature. The annotation of each metabolite and the level of identification were given based on the recommendations published by the chemical analysis working group metabolomics standards initiative [50].

### Metagenomic sequencing and read QC

To examine the gut microbiome of our lung cancer cohort, fecal samples were collected from 31 lung cancer patients at diagnosis, before the initiation of oncotherapy (baseline). Bacterial DNA was extracted using MO BIO PowerMax Soil DNA Extraction Kits (MO BIO Laboratories, Inc.) and purified with PowerClean Pro DNA Clean-Up Kits (MO BIO Laboratories, Inc.) according to the manufacturer’s protocol. Library preparation and shotgun metagenomic sequencing for all samples were performed by the Beijing genome institute using Illumina HiSeq 4000 with PE150 at an average depth of 6 Gb. The sequenced reads were processed with QC to remove the adapter regions, low-quality reads, and human DNA contaminations (bwa (version 0.7.4-r385) *mem* against human reference genome ucsc.hg19) following the previously described steps [51]. Approximately 95% of the reads remained after the QC.

The 471 metagenomic data from the 500FG project were used as European healthy control in the taxa comparison [25]. The taxonomic profiles of these 500FG samples were acquired by using the R package *curatedMetagenomicData* (R 3.5.1, curatedMetagenomicData 1.13.3 package) [52].

### Microbial taxonomic profiling and community diversity analysis

The high-quality reads were taxonomically profiled using MetaPhlAn2 [53] with default settings. The differentially abundant taxa were identified using the Wald test implemented in the R package DESeq2 [54] v1.22.2 on the unrarefied relative abundance data, and the statistical significance was filtered with *p* < 0.05 unless otherwise stated. The alpha-diversity (Shannon index) of each sample and beta-diversities (Bray–Curtis dissimilarities) among samples were calculated with VEGAN (v2.5.3) [55] based on rarefied data. Rarefaction was applied to the abundance table in estimated mapped reads to the depth of the less abundant sample in order to equalize the depth among the samples. To test the difference in the microbial composition between two or more groups, ANOSIM (analysis of similarities) was employed based on the Bray–Curtis dissimilarity. For *Faecalibacterium prausnitzii* strain abundance comparison, the high-quality reads were further taxonomically classified by using Kaiju [56], which is a protein-level classification tool, with the microbial subset of the NCBI BLAST non-redundant protein database *nr* was used.

### Assembly-free functional annotation

The high-quality reads after the QC were processed by using HUMAnN2 [57]. In the pipeline, the reads were mapped to the database of UniRef90 gene families, and then the gene families were regrouped to MetaCyc reactions and KEGG orthologs (KOs) for pathways annotation. The quantified pathway abundances in the units of RPKs (read per kilobase) were normalized to copies per million (CPM) units by the provided script for further analyses. KEGG pathway enrichment analysis was performed using GAGE [33].

### De novo assembly and CAZy annotation

The high-quality reads after the QC were further assembled using IDBA-UD [58] with k-mer size ranging from 20 to 150 bp. The coding DNA sequence (CDS) regions were predicted using MetaGeneMark [59] with the default parameters. The predicted peptide sequences were mapped to the dbCAN database [60] using DIAMOND [61] with the default parameters for CAZy annotation. The abundance of genes was quantified with RPKM (reads per kilobase of transcript per million mapped reads).

### Classifier model

A random forest model was built and trained by performing five-fold cross-validation using an R package, caret (R 3.3.0, caret 6.0.81 package) based on the predictors of the differentially abundant bacterial species (*p* < 0.05) and MetaCyc pathways (*p* < 0.05) that were identified by comparing cachexia and non-cachexia patient groups. The model performance was evaluated using the area under the ROC curve (AUC). For external validation of the classifier, seven additional stool samples were obtained from US lung cancer patients (cachexia *n* = 2, non-cachexia *n* = 5) and were processed for metagenomics sequencing following the same protocol as for the training cohort.

### Statistical analysis

All statistical analyses were conducted in R software (R 3.3.0). The student’s *t*-test was used for normally distributed clinical data and metabolites levels, whereas Fisher’ test was used to compare categorical variables. For other continuous data that were not normally distributed, a non-parametric Wilcoxon rank-sum test was employed. Statistically different taxa were identified with the Wald test using R package DESeq2 (v1.22.2) on the unrarefied relative abundance data. Two-tailed *p*-values < 0.05 were considered significant unless otherwise stated. Multiple hypothesis testing corrections were based on the FDR [62].

## Supplementary information

Supplementary Information

## Data Availability

The shotgun metagenomic sequencing data have been deposited in the NCBI Sequence Read Archive (SRA) under accession number PRJNA626477.
