# Supplementary material for: Distinct composition and metabolic functions of human gut microbiota are associated with cachexia in lung cancer patients
Source: ISME J. 2021 May 17;15(11):3207–20. doi: 10.1038/s41396-021-00998-8 (PMC8528809; doi:10.1038/s41396-021-00998-8)
Supplement: Supplementary file 1 — Supplementary Information [file 41396_2021_998_MOESM1_ESM.docx]

Supplementary Information

**Distinct composition and metabolic functions of human gut microbiota are associated with cachexia in lung cancer patients**

Yueqiong Ni^1*^, Zoltan Lohinai^2*^, Yoshitaro Heshiki^1,3^, Balazs Dome^2^, Judit Moldvay^2^, Edit Dulka^4^, Gabriella Galffy^4^, Judit Berta^2^, Glen J. Weiss^5^, Morten O. A. Sommer^6^, Gianni Panagiotou^1,3,7,#^

^*^ These authors contributed equally to this work

**^#^**Corresponding author. Email: [gianni.panagiotou@leibniz-hki.de](mailto:gianni.panagiotou@leibniz-hki.de)

**This PDF file includes:**

Figures. S1 to S8

Tables S1-S3

**Fig. S1 Significantly differential abundant plasma metabolites in cachexia vs. non-cachexia group (*p*<0.05, Student’s t-test).** Orange: higher in cachexia; green: higher in non-cachexia. #, identified metabolites that are significantly different after FDR correction (FDR-corrected *p*<0.2).

**Fig. S2 Comparison of plasma amino acids in cachexia vs. non-cachexia group**. The x-axis indicates the difference of average metabolite levels (normalized) between two groups. Asterisks indicate significantly differential abundant metabolites (*p*<0.05, Student’s t-test). Orange: higher in cachexia; green: higher in non-cachexia.

**Fig. S3 Significantly differential abundant gut microbial species in cachexia vs. non-cachexia group using DESeq2 (*p*<0.05).** Color scale represents the row-scaled log-transformed relative abundances of species. Species annotated in orange: higher in cachexia; green: higher in non-cachexia.

**Fig. S4 The abundance comparison of *Faecalibacterium prausnitzii* strains between cachexia and non-cachexia patient groups.**


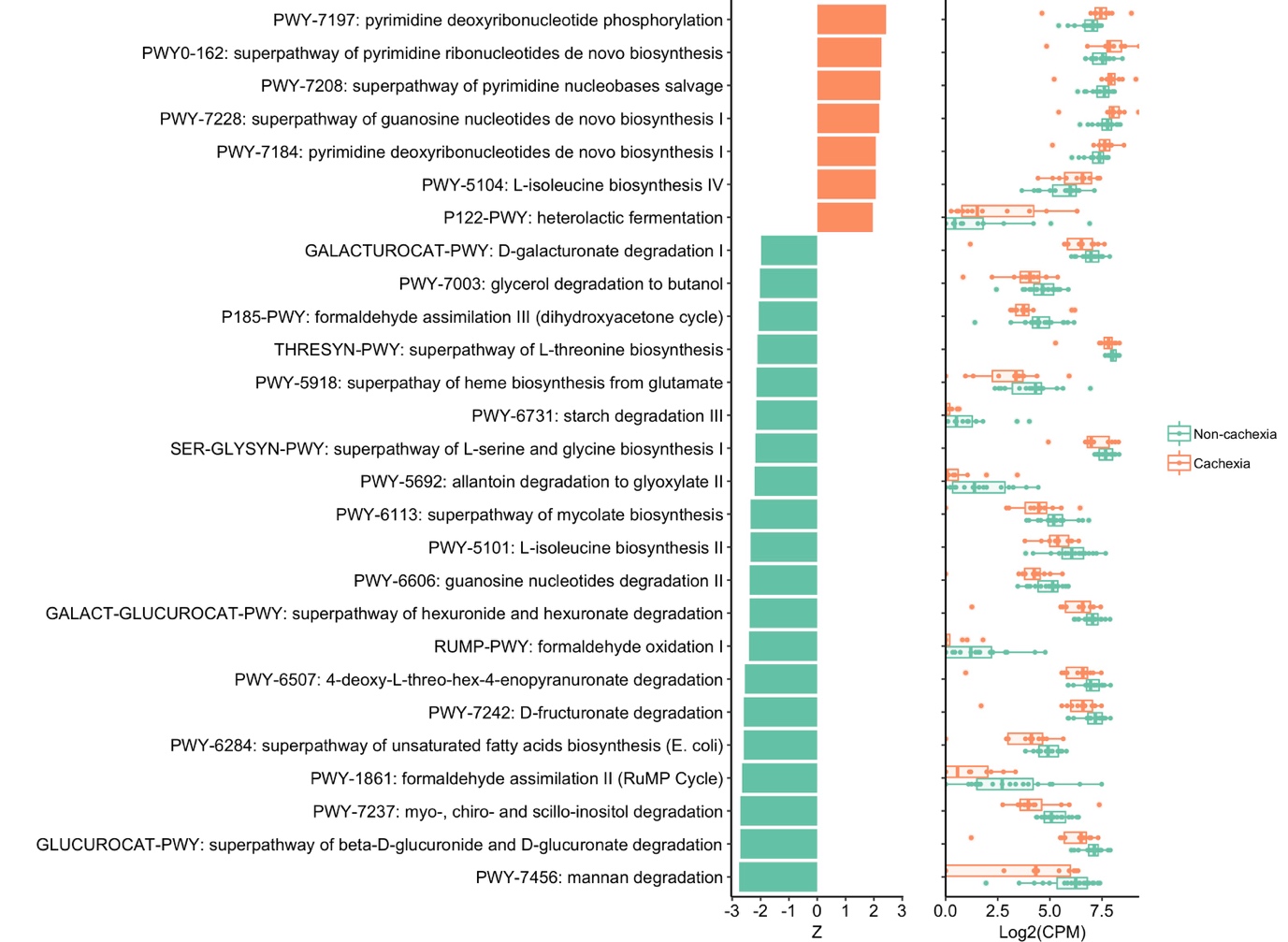


**Fig. S5 Significantly differential abundant MetaCyc pathways in cachexia vs. non-cachexia group (*p*<0.05, Wilcoxon rank-sum test).** Orange: higher in cachexia; green: higher in non-cachexia.

**
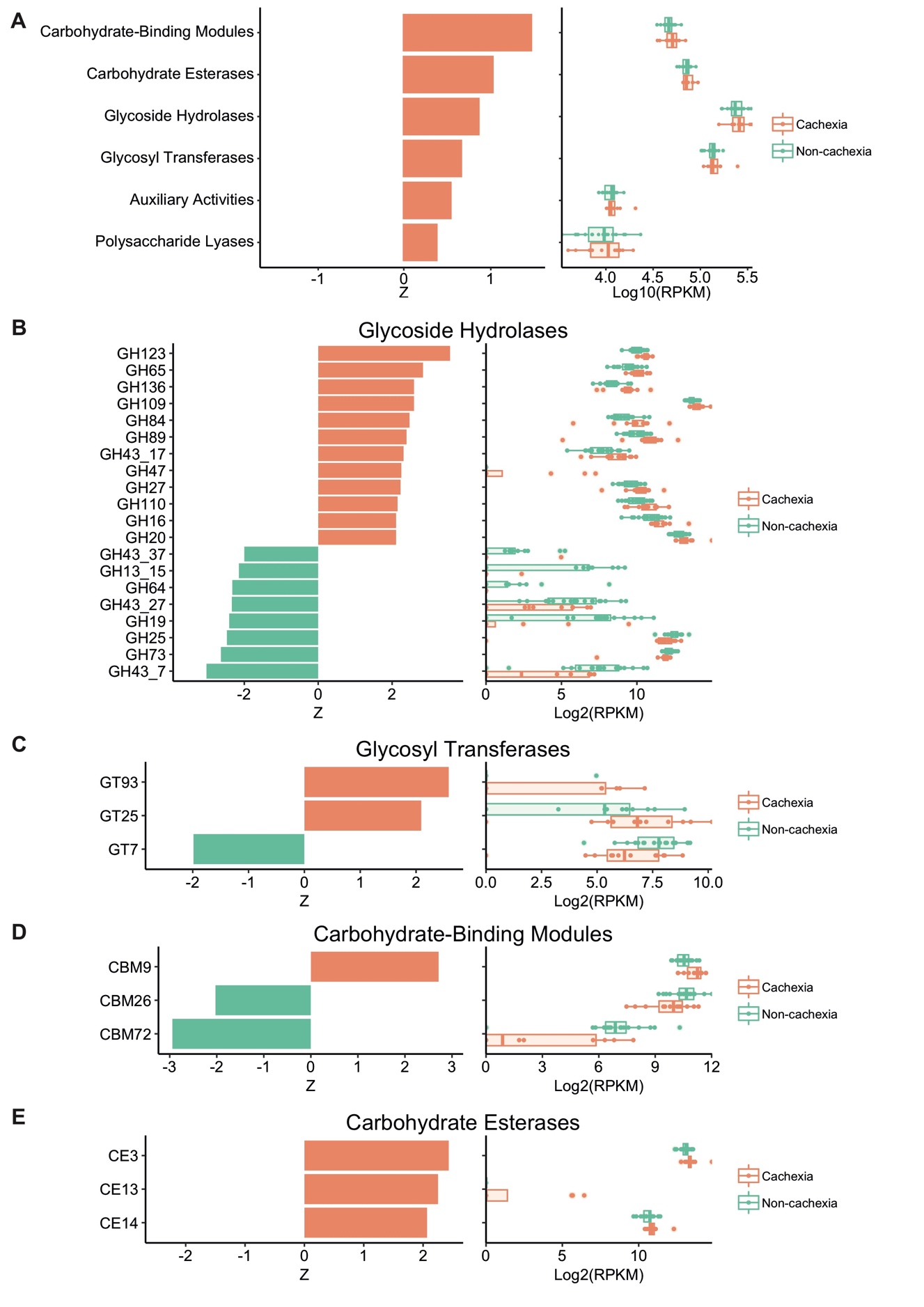
**

**Fig. S6 Comparison of CAZy abundances in cachexia vs. non-cachexia group**. (A) Overall comparison of CAZY class abundances. All CAZy classes were more abundant in the cachexia group, but none of them have significantly differences (*p*>0.05, Wilcoxon rank sum test). (B-E) Significantly differential abundant CAZy families within each CAZy class (*p*<0.05, Wilcoxon rank sum test). Orange: higher in cachexia; green: higher in non-cachexia.

**Fig. S7 Correlation between gut microbiota and plasma vitamins**. Heatmap of Spearman’s rank correlation analysis between significant microbiota features and vitamins (**p*<0.05, ***p*<0.01, ****p*<0.001, +FDR<0.1, ++FDR<0.05, +++FDR<0.01, Spearman’s rank correlations). Microbiota-derived features used here include significant species from DESeq2, differentially abundant MetaCyc pathways and significant KEGG pathways from enrichment analysis. Orange: higher in cachexia; green: higher in non-cachexia.

**Fig. S8** **List of top 50 feature importance for the random forest classifier using abundances of gut microbiota species (blue) and MetaCyc pathways (red).**

| **Supplementary Table 1. Major clinicopathological characteristics of the EU/Hungarian patients cohort (n=31).** | | | | | | | |
| --- | --- | --- | --- | --- | --- | --- | --- |
| **SampleID** | **Gender** | **Age** | **Stage*** | **Stage** | **Histology** | **Cachexia** | **SGA** |
| 03MT1 | M | 61 | NA | Advanced | ADC | Non-Cachexia | A |
| 06MT1 | F | 63 | IVA | Advanced | ADC | Cachexia | B |
| 14MT1 | F | 69 | IIIB | Advanced | SCC | Cachexia | B |
| 18MK1 | M | 69 | IVA | Advanced | ADC | Non-Cachexia | A |
| 22MT1 | M | 64 | IVA | Advanced | SCC | Cachexia | C |
| 29MT1 | F | 60 | IB | Advanced | SCC | Cachexia | C |
| 31MT1 | F | 70 | IVA | Advanced | ADC | Non-Cachexia | A |
| 35MT1 | F | 51 | IIIB | Advanced | SCLC | Non-Cachexia | A |
| 36MT1 | F | 60 | IVB | Advanced | NSCLC-NOS | Cachexia | B |
| 41MT1 | M | 67 | IVA | Advanced | SCC | Cachexia | B |
| 45MT1 | M | 65 | IIIA | Advanced | SCLC | Non-Cachexia | A |
| 51MT1 | F | 67 | IA | Advanced | SCC | Cachexia | B |
| 55MK1 | M | 67 | IIA | Advanced | ADC | Non-Cachexia | A |
| 60MT1 | M | 64 | IVB | Advanced | SCC | Non-Cachexia | A |
| 61MK1 | M | 64 | IIB | Advanced | SCLC | Non-Cachexia | A |
| 61MT1 | M | 70 | IVC | Advanced | SCC | Non-Cachexia | A |
| 64MT1 | M | 63 | IVA | Advanced | ADC | Cachexia | C |
| 70MK1 | F | NA | IIIA | Advanced | SCC | Non-Cachexia | A |
| 73MK1 | M | 64 | IIIB | Advanced | SCC | Cachexia | B |
| 75MK1 | F | 63 | IVB | Advanced | SCLC | Non-Cachexia | A |
| 77MK1 | M | 68 | IVA | Advanced | ADC | Non-Cachexia | A |
| 83MK1 | M | 72 | IIIB | Advanced | ADC | Cachexia | C |
| 90MK1 | M | 78 | IVA | Advanced | ADC | Non-Cachexia | A |
| 04MK1 | M | 50 | IIIA | Resected | SCC | Non-Cachexia | A |
| 09MT1 | M | 75 | IB | Resected | ADC | Non-Cachexia | A |
| 10MK1 | F | 65 | IIA | Resected | ADC | Cachexia | B |
| 11MT1 | F | 57 | IIA | Resected | ADC | Cachexia | B |
| 12MK1 | M | 63 | IIA | Resected | ADC | Non-Cachexia | A |
| 20MT1 | M | 57 | IB | Resected | ADC | Non-Cachexia | A |
| 58MT1 | M | 68 | IVA | Resected | ADC | Non-Cachexia | A |
| 82MK1 | F | 51 | IIB | Resected | ADC | Non-Cachexia | A |
| *according to the Union for International Cancer Control (8th edition) | | | | | | | |
| adenocarcinoma (ADC), squamous cell carcinoma (SCC), non-small cell lung carcinoma not otherwise specified (NSCLC-NOS), small cell lung carcinoma (SCLC) | | | | | | | |

**Supplementary Table 2.** **Comparison of main anthropometric and clinical characteristics of the EU/Hungarian patients cohort**.

|  | Cachexia  (n=12) | Non-Cachexia  (n=19) | *P*-value | | Used test |
| --- | --- | --- | --- | --- | --- |
| **Age** |  |  | 0.9557 | Student’s t test | |
| Mean | 64.25 | 64.11 |  |  | |
| SD | 4.16 | 7.86 |  |  | |
| **Gender** |  |  | 0.1304 | Fisher's exact | |
| Male | 5 | 14 |  |  | |
| Female | 7 | 5 |  |  | |
| **Antibiotics usage** |  |  | 0.5097 | Fisher's exact | |
| Yes | 0 | 2 |  |  | |
| No | 12 | 17 |  |  | |
| **PPI blocker usage** |  |  | 1 | Fisher's exact | |
| Yes | 4 | 7 |  |  | |
| No | 8 | 12 |  |  | |
| **Type 2 diabetes** |  |  | 1 | Fisher's exact | |
| Yes | 1 | 2 |  |  | |
| No | 11 | 17 |  |  | |
| **NAFLD** |  |  | 0.5097 | Fisher's exact | |
| Yes | 0 | 2 |  |  | |
| No | 12 | 17 |  |  | |

| **Supplementary Table 3. Major clinicopathological characteristics of the US patients cohort (n=7).** | | | | | | | | |
| --- | --- | --- | --- | --- | --- | --- | --- | --- |
| **PatientID** | **Gender** | **Age** | **Race** | **Stage*** | **Stage** | **Histology** | **Cachexia** | **SGA** |
| 01A1 | F | 53 | Black | IV | Advanced | ADC | Non-Cachexia | A |
| 05A1 | F | 54 | White | IV | Advanced | ADC | Cachexia | B |
| 07A1 | M | 53 | White | IV | Advanced | ADC | Non-Cachexia | A |
| 08A1 | F | 51 | White | IV | Advanced | ADC | Non-Cachexia | A |
| 09A1 | F | 52 | White | IV | Advanced | ADC | Non-Cachexia | A |
| 12A1 | M | 64 | Hispanic | IV | Advanced | ADC | Non-Cachexia | A |
| 25A1 | M | 60 | White | IV | Advanced | SCLC | Cachexia | B |
| *according to the Union for International Cancer Control (8th edition) | | | | | | | | |
| adenocarcinoma (ADC), squamous cell carcinoma (SCC), non-small cell lung carcinoma not otherwise specified (NSCLC-NOS), small cell lung carcinoma (SCLC) | | | | | | | | |
